# SOCS1 as a Biomarker Candidate for HPV Infection and Prognosis of Head and Neck Squamous Cell Carcinomas

**DOI:** 10.3390/cimb45070353

**Published:** 2023-06-30

**Authors:** Manli Guo, Lijie Zhang, Huihui Wang, Qiaozhen Zhou, Xinrang Zhu, Xinyu Fu, Jinlong Yang, Shanhe Liu, Dingcheng Guo, Baoping Zhang

**Affiliations:** 1 Key Lab of Oral Diseases of Gansu Province, Key Laboratory of Stomatology of State Ethnic Affairs Commission, Northwest Minzu University, Lanzhou 730030, China; guoml@xbmu.edu.cn; 2 School/Hospital of Stomatology, Lanzhou University, Donggang West Road 199, Lanzhou 730000, China; ljzhang18@lzu.edu.cn (L.Z.); lzu_wanghuihui@lzu.edu.cn (H.W.); zhouqzh20@lzu.edu.cn (Q.Z.); zhuxr20@lzu.edu.cn (X.Z.); fuxy19@lzu.edu.cn (X.F.); yangjl2019@lzu.edu.cn (J.Y.); liushh2019@lzu.edu.cn (S.L.); guodch21@lzu.edu.cn (D.G.); 3 Key Laboratory of Dental Maxillofacial Reconstruction and Biological Intelligence Manufacturing, Lanzhou University, Lanzhou 730000, China; 4 Institute of Biomechanics and Medical Engineering, Lanzhou University, Lanzhou 730000, China; 5 Key Laboratory of Mechanics on Disaster and Environment in Western China, Ministry of Education, College of Civil Engineering and Mechanics, Lanzhou University, Lanzhou 730000, China

**Keywords:** head and neck squamous cell carcinoma (HNSCC), human papillomavirus (HPV), TCGA database, differential gene, suppressor of cytokine signaling 1 (SOCS1)

## Abstract

The pathogenesis of head and neck squamous cell carcinoma (HNSCC) is associated with human papillomavirus (HPV) infection. However, the molecular mechanisms underlying the interactions between HNSCC and HPV remain unclear. Bioinformatics was used to analyze the gene expression dataset of HPV-associated HNSCC based on the Cancer Genome Atlas (TCGA) database. Differentially expressed genes (DEGs) in HPV-positive and HPV-negative HNSCC were screened. Gene function enrichment, protein–protein interactions (PPI), survival analysis, and immune cell infiltration of DEGs were performed. Furthermore, the clinical data of HNSCC tissue samples were analyzed using immunohistochemistry. In total, 194 DEGs were identified. A PPI network was constructed and 10 hub genes (EREG, PLCG1, ERBB4, HBEGF, ZFP42, CBX6, NFKBIA, SOCS1, ATP2B2, and CEND1) were identified. Survival analysis indicated that low expression of SOCS1 was associated with worse overall survival. Immunohistochemistry demonstrated that SOCS1 expression was higher in HPV-negative HNSCC than in HPV-positive HNSCC, and there was a positive correlation between SOCS1 expression and patient survival. This study provides new information on biological targets that may be relevant to the molecular mechanisms underpinning the occurrence and development of HNSCC. SOCS1 may play an important role in the interaction between HPV and HNSCC and serve as a potential biomarker for future therapeutic targets.

## 1. Introduction

Head and neck cancers (HNCs) are a group of malignant tumors originating from the oral cavity, oropharynx, hypopharynx, and larynx [1]. According to Global Cancer Statistics 2020, approximately 745,000 people are diagnosed with HNC worldwide, making HNC the eighth most common malignancy [2]. Head and neck squamous cell carcinoma (HNSCC) is the most prevalent pathological type of HNC, which is strongly associated with heavy use of tobacco and alcohol, as well as genetics, exposure to toxic substances, diet, and the environment [3,4,5]. Human papillomavirus (HPV) infection, particularly with the HPV16 subtype, is also a key factor in the development of HNSCC [6]. There has been a marked increase in the incidence of HPV-positive HNSCC which now represents the most common form of oropharyngeal cancer in many western populations [7,8]. HPV-positive HNSCC is associated with improved prognosis compared with HPV-negative HNSCC [9,10,11]. However, the exact mechanism through which HPV infection is involved in the development of HNSCC remains unclear. Many patients with HNSCC present with locally advanced disease, frequently with prominent involvement of the lymph nodes, which is associated with a poor prognosis [12]. Therefore, it is vital to identify biomarkers for early diagnosis of HNSCC to improve treatment and prognosis.

The suppressor of cytokine signaling (SOCS) family of proteins comprises negative-feedback inhibitors of signaling induced by cytokines that act via the JAK/STAT pathway [13]. SOCS1, the most potent member, has been the most extensively discussed. SOCS1 has been found to be silenced in primary tumors in >50% of hepatocellular carcinoma [14], 44% of gastric carcinoma [15], 75% of melanoma [16], 40% of hepatoblastoma cases, 53–71% of pancreatic cancers and solid tumors, and in 60% of acute myeloid lymphoma [17] and 62% of multiple myeloma [18]. However, the correlation between SOCS1 expression and HNSCC has not been previously explored in depth.

Gene expression analysis driven by high-throughput data has become an increasingly meaningful tool for examining the biological mechanisms of diseases as a result of the quick development of high-throughput technologies such as RNA sequencing [19]. The Cancer Genome Atlas (TCGA) offers a wealth of cancer-related high-throughput sequence datasets that enable a thorough understanding of cancer genetics through cataloging all potential cancer drivers, identifying robust prognostic and predictive biomarkers, as well as new druggable therapeutic targets [20]. In this study, RNA-seq data and clinical information about HNSCC were mined and collated from TCGA database to obtain an HNSCC sample library containing complete RNA-seq data coupled with information on HPV status. Through bioinformatic analysis of the HNSCC sample base, we aim to investigate the molecular regulatory mechanisms related to the occurrence of HPV and HNSCC and develop a new direction in bioinformatics analysis that may be useful in the prevention, diagnosis, and treatment of HPV-related head and neck tumors.

## 2. Materials and Methods

### 2.1. Data Collection and Processing

RNA-seq data and clinical information on HNSCC were obtained from TCGA database. After sorting the HNSCC sample data, eight samples that contained incomplete data were eliminated, leaving 521 samples that contained both RNA-seq data and the corresponding clinical information. For the 521 samples, the dplyr function of R software (v 1.0.4) was used to screen for samples containing HPV status information, and HPV-positive and HPV-negative samples were selected for further analysis.

### 2.2. Analysis of Differentially Expressed Genes (DEGs)

The annotations that were obtained from TCGA gene expression matrix were converted into standard gene names with official gene symbols using the cluster profiler of R software (v 3.18.1) and org.Hs.eg.db (v 3.12.0). Furthermore, the gene expression matrix was converted to a standardized gene expression matrix file to facilitate differential gene expression analysis.

The DEGs between the HPV fluorescence in situ hybridization (FISH)-positive sample group and HPV FISH-negative sample group were analyzed using the limma package of R software (v 3.46.0). The log2 fold change (log2 FC) and *p* value for each gene were also obtained. The screening criteria for DEGs were *p* < 0.05 and |log2 FC| ≥ 0.6. In order to evaluate the diagnostic value of these top ten genes, we evaluated their diagnostic value using the ROC curve.

### 2.3. KEGG Pathway and GO Function Enrichment Analysis

To predict the biological function of DEGs between the HPV-positive and the HPV-negative samples further, the KOBAS-i database (http://kobas.cbi.pku.edu.cn (accessed on 6 June 2023)) [21] and Gene Ontology (GO; http://geneontology.org/ (accessed on 6 June 2023)) [22] were used to carry out Kyoto encyclopedia of genes and genomes (KEGG) pathway and GO function enrichment analyses. The screening thresholds were set to count > 2 and *p* < 0.05. The ggplot2 package in R software (v 3.3.3) was employed to visualize all important KEGG signaling pathways and GO biological processes.

### 2.4. Protein–Protein Interaction Analysis of DEGs and Hub Genes Screening

DEGs were imported into the STRING database for analysis of protein–protein interactions (PPI) [23]. The lowest interaction score was set to the medium confidence level (0.400) to obtain data on the interactions between proteins encoded by DEGs. PPI were visualized using Cytoscape (v 3.8.2) to create a functional protein association network. In conjunction with this, hub genes with high connectivity in the associated network were identified using the CytoHubba plug-in, based on the top 10 results of the dependable maximum net capability (DMNC) algorithm.

### 2.5. Survival Analysis of Hub Genes

Gene expression profiling interactive analysis (GEPIA) was used to investigate the relationship between hub gene expression and HNSCC prognosis [24]. The survival curves of the hub gene in patients with HNSCC were plotted according to the survival analysis of hub genes screened using GEPIA. Statistical significance was set at *p* < 0.05.

### 2.6. Single Sample Gene Set Enrichment Analysis (ssGSEA)

The gene expression profile of a single sample can be changed into a gene set enrichment profile using ssGSEA. The density of immune cells that infiltrate tumors is represented by the enrichment score of a gene set associated with immune cells [25].

The gene set specific to the immune cell population was obtained from a molecular feature database. The GSVA package of R software (v 1.38.2) was used to characterize and quantify the difference in infiltrating immune cells between the HPV-positive and HPV-negative sample groups from the gene expression data of patients with head and neck cancer and to determine the difference in infiltration of different immune cell populations. Metabolic pathway gene sets were obtained from a molecular signature database. Using the GSVA package of R, the metabolic differences between the HPV-positive and HPV-negative sample groups were explored.

### 2.7. Tissue Preparation

The study protocol was approved by the Ethics Committee of First Hospital of Lanzhou University (No. LDYYLL-2022-321). HNSCC tissue samples were collected from 36 patients at the Department of Oral and Maxillofacial Surgery, First Hospital of Lanzhou University, after obtaining written informed consent. All specimens obtained from a typical area of the lesion were analyzed by two qualified pathologists to rule out additional diseases, fixed in 10% neutral-buffered formalin, and embedded in paraffin.

### 2.8. Immunohistochemistry

The expression of SOCS1 in the hematoxylin and eosin-stained sections was detected using the SP method. The sections were cut into 4 μm pieces and fixed in 4% neutral paraformaldehyde in 0.1 M phosphate buffer. They were then paraffin-embedded, dewaxed, and hydrated with gradient ethanol. This was followed by incubation with rabbit anti-human polyclonal SOCS1 antibody (ab137384 at 1/100 dilution, Abcam) at 4 °C and then with a secondary antibody (HistostainTM-Plus Kits, SP-9000) at 37 °C for 1 h. 3, 3′-diaminobenzidine (DAB, Zhongshan Jinqiao Biotechnology Co., Ltd., Beijing, China) was used during dehydration, tissue clearing, and neutral resin sealing. An Olympus BX53 microscope was used to visualize staining.

### 2.9. Statistical Analysis

Statistical analysis was performed using SPSS 26.0 (IBM, Armonk, NY, USA). The data were represented as mean with standard error and statistically analyzed using one-way ANOVA. The correlation between clinical features and HPV status was examined using Pearson’s chi-square test based on computed odds ratios and 95% confidence intervals. The significance level was chosen at *p* < 0.05.

## 3. Results

### 3.1. Analysis of DEGs

**Figure 1:**
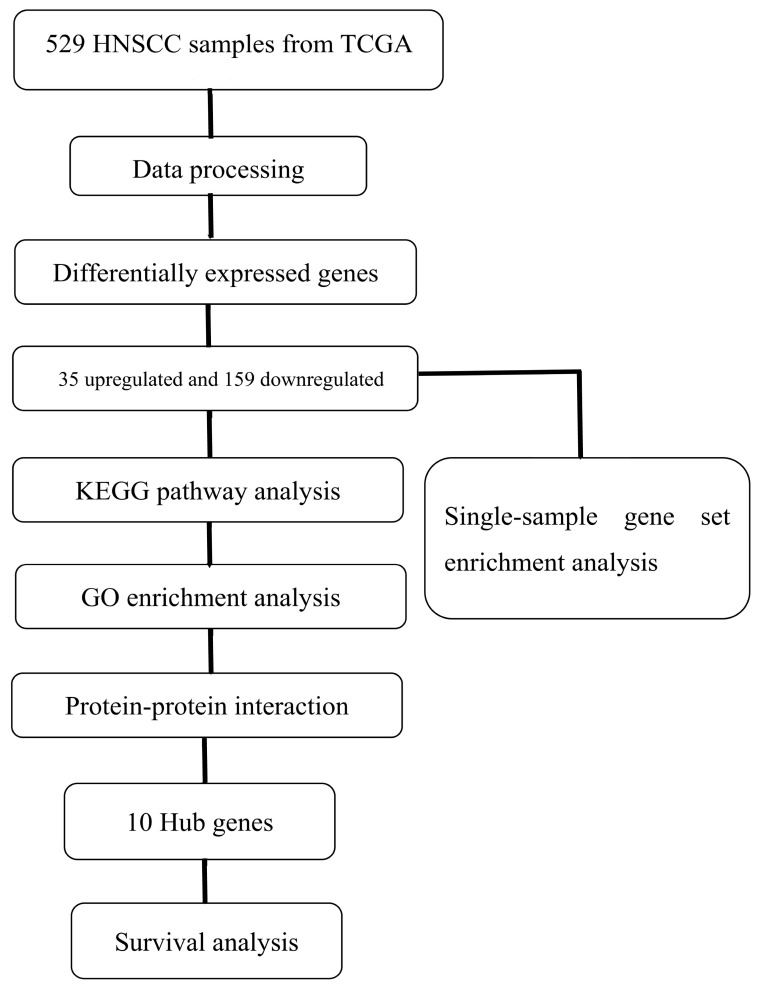
Flow chart of the study. TCGA, The Cancer Genome Atlas; HNSCC, Head and Neck Squamous Cell Carcinoma; KEGG, Kyoto Encyclopedia of Genes and Genomes; GO, Gene Ontology.

A flow chart of the bioinformatic analysis is displayed in Figure 1. The RNA-seq data and clinical information of HNSCC obtained from TCGA database included 529 tumor samples. After sorting the HNSCC sample data, 521 samples containing RNA-seq data and the corresponding clinical information were included. The dplyr function of R software was then used to select the samples containing HPV status information, leading to 86 samples being retained. Among these, there were 21 HPV FISH-positive samples and 65 HPV FISH-negative samples.

**Figure 2:**
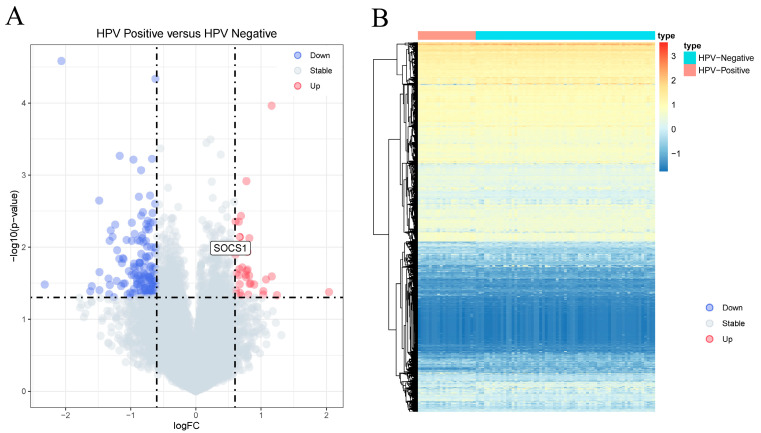
Analysis of differentially expressed genes. (**A**) Volcano plot of differential genes between HPV-positive sample group and HPV-negative sample group. The *X*-axis represents the log2 fold change, and the *Y*-axis represents the −log10 (*p* value). (**B**) Heat map; red indicates that the gene expression level is increased, blue indicates that the gene expression level is decreased, purple indicates HPV-negative sample group, and green indicates HPV-positive sample group.

Analysis of the HPV-positive and HPV-negative samples revealed 194 DEGs. These included 35 upregulated and 159 downregulated DEGs (|log2 FC| ≥ 0.6, *p* < 0.05) (Supplementary Table S1). The top ten upregulated DEGs were XIST, TSIX, C3orf57, CRYBB2, FAM153A, LCE1A, TAGLN3, FTCD, PAX6 and C1orf51, and the ten most downregulated DEGs were C20orf114, GATSL2, ZFY, PRKY, CECR2, NLGN4Y, MSMB, SERPINA3, SRL and ESRRG (Supplementary Table S2). Volcanic and heat maps in Figure 2A,B show the results of gene analysis for differences between HPV-positive and HPV-negative samples, respectively. The ROCR package in R software (v 4.3.0) was utilized to calculate the AUC values and plot the ROC curves of the hub gene in patients with HNSCC, we found that XIST, TSIX, PAX6, FAM153A, CRYBB2, C1orf51, and C3orf57 exhibited AUC values ranging from 0.5 to 0.7, indicating a relatively lower predictive value. The remaining three genes in the top ten had AUC values of less than 0.5. ZFY, CECR2, MSMB, NLGN4Y, and PRKY were observed to have AUC values between 0.5 and 0.7, suggesting a lower predictive value. Conversely, the AUC values of the remaining five genes among the top ten downregulated genes were less than 0.5 (Supplementary Figures S1 and S2).

### 3.2. KEGG and GO Analysis of DEGs

**Figure 3:**
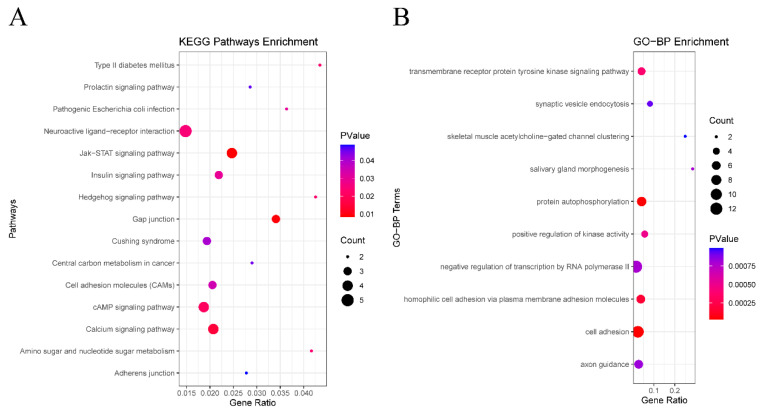
Functional enrichment of analysis of DEGs based on GO and KEGG. (**A**) The top 10 terms in the GO-BP category of GO analyses DEGs. (**B**) KEGG pathway analysis of DEGs.

KEGG pathway enrichment analysis was performed on the selected DEGs, with the results demonstrating that the DEGs were significantly enriched in 15 signaling pathways, including those related to type II diabetes mellitus, prolactin signaling pathway, pathogenic Escherichia coli infection, and others (Supplementary Table S3, Figure 3A). Furthermore, our analysis revealed that ATP2B2, CLDN1, EGFR, SOCS7, and other genes were among the top pathways identified. However, no statistical significance was demonstrated in their survival curves (*p* > 0.05) (Supplementary Figure S3). Additionally, NLGN4Y, which is among the top ten downregulated genes, has been implicated in cell adhesion molecules (CAMs).

A total of 111 BP terms, 41 CC terms, and 53 MF terms were enriched via the GO system. In this study, which focused on BP categories, we found that these DEGs were closely related to the transmembrane receptor protein tyrosine kinase signaling pathway, synaptic vesicle endocytosis, homophilic cell adhesion via plasma membrane adhesion molecules, skeletal muscle acetylcholine-gated channel clustering, and salivary gland morphogenesis (Supplementary Table S4), suggesting that HPV infection may affect the prognosis of HNSCC through interfering with protein metabolism and cell adhesion. Figure 3B shows the top 10 terms in the GO-BP category. Several genes such as CDHR4, DCHS2, SYT2, and PCDHGA5 were included in the significantly enriched BP, among which only CDHR4 and NLGN4Y were statistically significant (*p* < 0.05) (Supplementary Figure S4). Among the top ten upregulated genes and the top ten downregulated genes, TAGLN3 was included in negative regulation of transcription by RNA polymerase II, and NLGN4Y was involved in synaptic vesicle endocytosis. PAX6 was involved in negative regulation of transcription by RNA polymerase II, salivary gland morphogenesis, and axon guidance.

### 3.3. PPI Network Construction and Screening for Hub Genes

**Figure 4:**
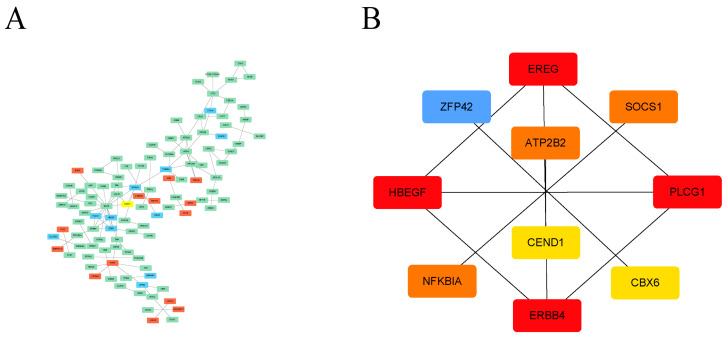
PPI analysis of DEGs based on Cytoscape. (**A**) Visualized PPI analysis of DEGs; red indicates that the gene expression level is increased, green indicates that the gene expression level is decreased, and blue indicates that the gene expression level has no significant difference. (**B**) Interconnections of 10 hub genes; darker color represents higher DMNC scores.

Additionally, we explored the interaction relationships of 194 DEGs using the STRING tool. The confidence was set to 0.4, and after removing discrete proteins, we could visualize a PPI network containing 108 nodes and 110 edges (Figure 4A). The CytoHubba plug-in was adopted to analyze the network, and the top 10 scoring genes were screened as hub genes using the DMNC algorithm (Figure 4B, Supplementary Table S5). The ten hub genes were EREG, PLCG1, ERBB4, HBEGF, ZFP42, CBX6, NFKBIA, SOCS1, ATP2B2, and CEND1 (Table 1).

### 3.4. Survival Analysis of Hub Genes

**Figure 5:**
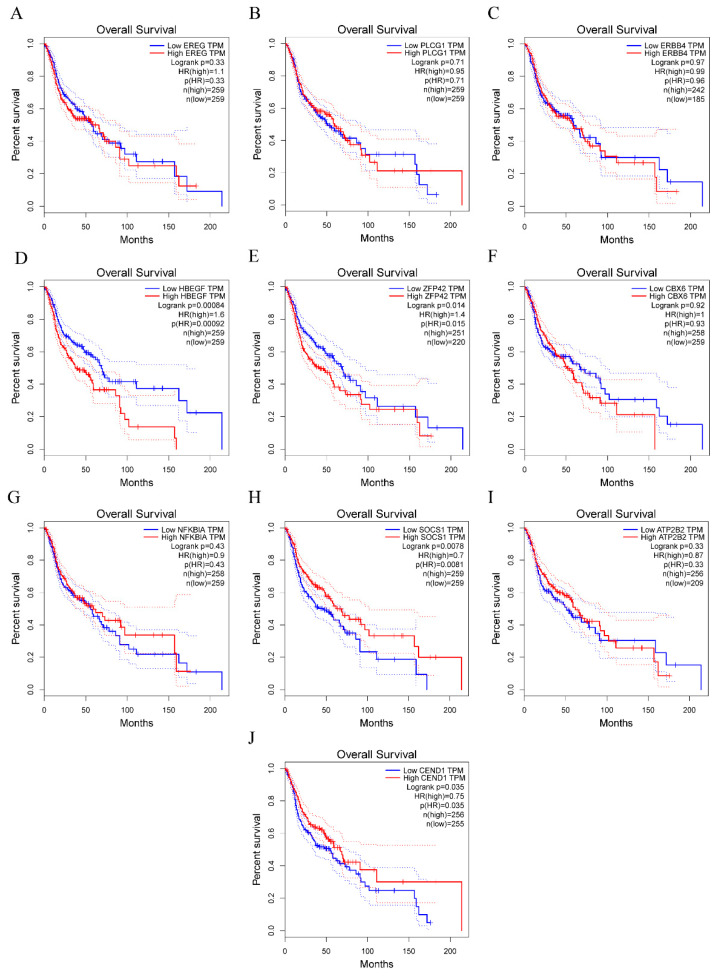
Survival analysis for hub genes. (**A**–**J**) show overall survival rate of ten hub genes.

**Figure 6:**
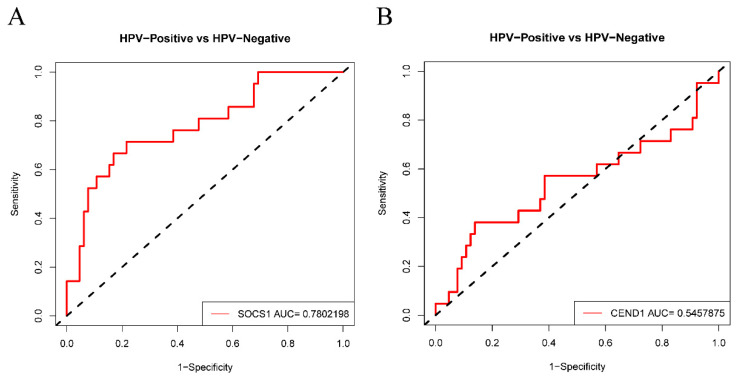
Diagnostic value of SOCS1 and CEND1. (**A**) ROC analysis of SOCS1. (**B**) ROC analysis of CEND1.

The results of the GEPIA analysis indicated that elevated expression levels of SOCS1 and CEND1 were significantly correlated with increased overall survival rates in comparison to diminished expression levels of these genes (*p* < 0.05). Conversely, heightened expression levels of HBEGF and ZFP42 were significantly associated with decreased overall survival rates in comparison to reduced expression levels of these genes (*p* < 0.05). However, the expression levels of the remaining six hub genes did not demonstrate a significant correlation with the prognosis of HNSCC (*p* > 0.05) (Figure 5). Therefore, there is a statistically significant association between overall survival and SOCS1 expression in patients with HNSCC. ROC curves were drawn to assess the predictive value of SOCS1 and CEND1 for HPV infection. The results showed that the area under the curve (AUC) for SOCS1 was 0.7802198, indicating a superior diagnostic capacity in comparison to CEND1 (Figure 6).

### 3.5. SsGSEA Results

**Figure 7:**
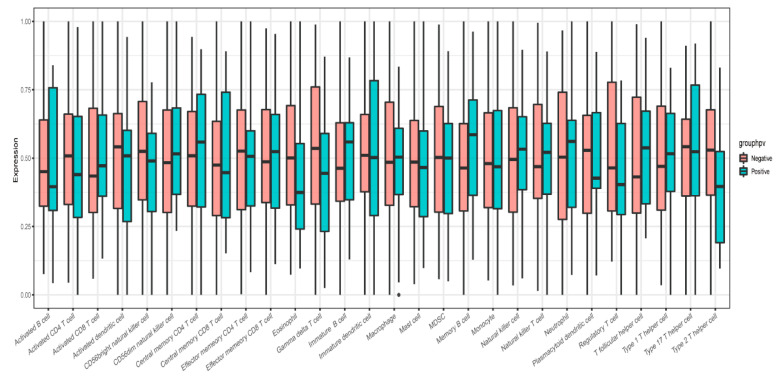
ssGSEA immunoassay indicated infiltration level of 28 immune cells in tumor immune microenvironment.

**Figure 8:**
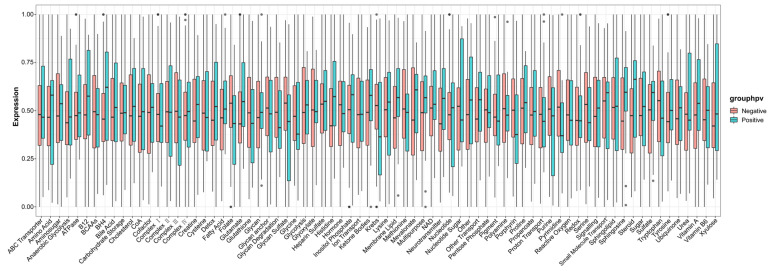
ssGSEA metabolic analysis revealed certain changes in metabolic pathways between HPV-positive and HPV-negative samples.

The infiltration levels of 28 immune cells in the tumor immune microenvironment were quantitatively analyzed using ssGSEA based on 28 immune markers in HPV-positive and HPV-negative samples. The results showed that the immune systems of HPV-positive HNSCC samples were not significantly different from those of HPV-negative samples (Figure 7). Metabolic analysis of HPV-positive and HPV-negative samples revealed certain changes in metabolic pathways, including histidine, pyrimidine, steroids, and membrane lipids (Figure 8).

### 3.6. Expression of SOCS1 in Different HNSCC Tissues and Patient Survival

**Figure 9:**
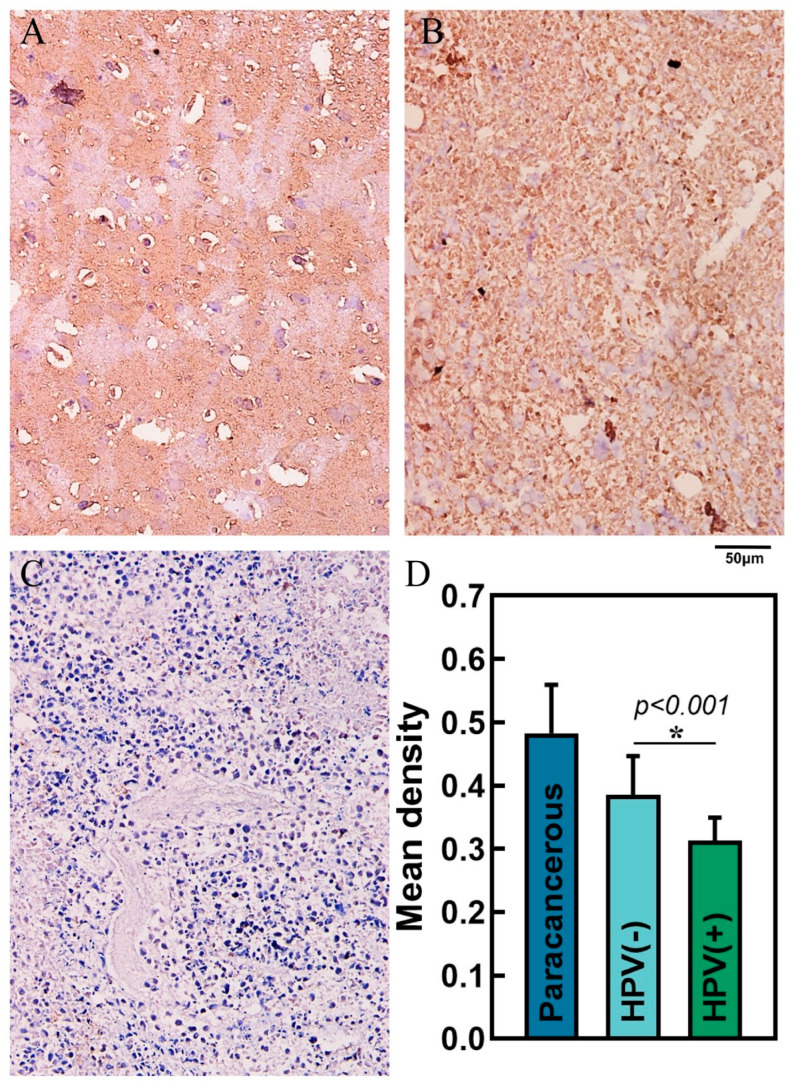
Immunohistochemistry and mean density in different tissues. (**A**) Paracancerous tissue, (**B**) HPV-negative HNSCC tissue, (**C**) HPV-positive HNSCC tissue—scale bar, 50 μm. (**D**) Mean density of paracancerous, HPV-negative HNSCC tissue and HPV-positive HNSCC tissue.

**Figure 10:**
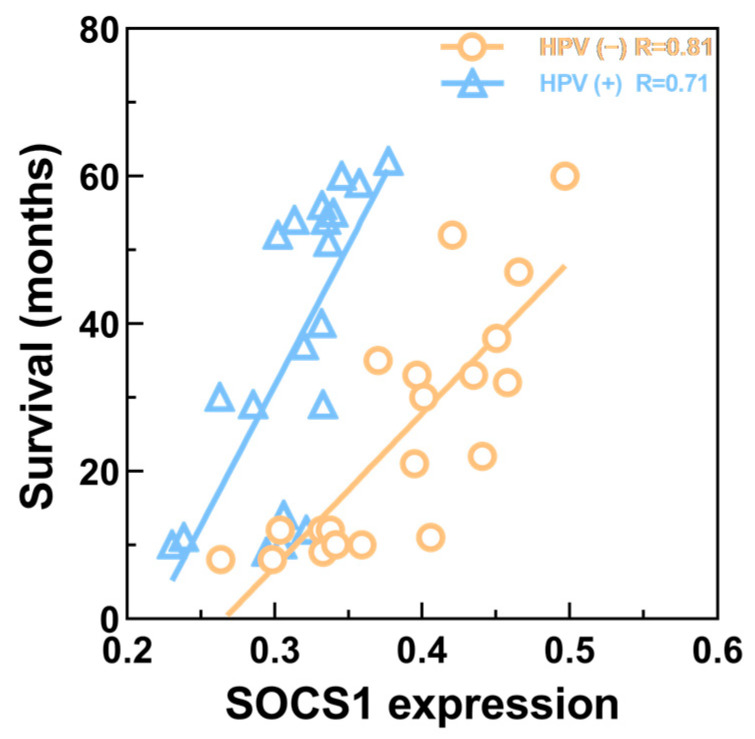
Correlation between SOCS1 expression and patient survival. R = 0.822 in HPV-positive HNSCC tissues; R = 0.843 in HPV-negative HNSCC tissues (*p* < 0.001).

The results demonstrated that the relationship between the expression of SOCS1 in different HNSCC tissues was as follows: paracancerous tissues > HPV-negative HNSCC tissues > HPV-positive HNSCC tissues (Figure 9). Their average densities were 0.48 ± 0.076, 0.39 ± 0.061, and 0.31 ± 0.036, respectively. The average survival months of high and low expression of SOCS1 was 26.4 ± 15.6 and 37.2 ± 19.1. Patient survival and SOCS1 expression levels were found to be positively correlated, according to the Pearson correlation coefficient (R = 0.843 in HPV-negative tissues, R = 0.822 in HPV-positive tissues, *p* < 0.001) (Figure 10).

## 4. Discussion

HNSCC is frequently invasive and is likely to recur after treatment, yet the early symptoms of HNSCC are typically not noticeable. Local progression of lymph node metastases has been observed in patients diagnosed with HNSCC [26,27]. The five-year survival rate of HNSCC is only 40–50% with poor clinical prognosis, with the disease seriously threatening the structure and function of facial tissues and health status [28,29]. A recent study has confirmed that HPV16 is a major risk factor for HNSCC development [30]. As an unenveloped circular double-stranded DNA virus, HPV can infect basal epithelial cells and induce benign and malignant lesions in the genital and upper digestive tracts, skin, and mucosa, which is conducive to the occurrence and development of tumors [31]. HPV integration affects the host genome and changes the expression of host genes through amplifying oncogenes, promoting the degradation of tumor suppressor proteins, and driving inter-chromosome or intra-chromosome rearrangements. Genome-wide sequencing of HPV-positive HNSCC tissues revealed that HPV-induced genomic alterations often disrupt the expression and structure of adjacent genes involved in tumorigenicity, leading to amplification and expression of viral oncogenes E6 and E7 [32]. The molecular mechanisms underlying the specific role of HPV in HNSCC were further investigated here.

In this study, we analyzed the gene expression data of HNSCC samples in relation to HPV infection status. A total of 300 upregulated DEGs and 481 downregulated DEGs were found in HPV-positive HNSCC samples compared to HPV-negative HNSCC samples. Among these DEGs, X Inactive Specific Transcript (XIST) was the most significantly upregulated. XIST is involved in cell differentiation, cell proliferation, and genome maintenance in human cells [33]. Abnormal XIST expression plays a vital role in the occurrence and development of many cancers [34]. Previous studies have shown that lncRNA XIST promotes the development of laryngeal squamous cell carcinoma (LSCC) through stimulating miR-144 to regulate the expression of IRS1; interference with its expression can inhibit the proliferation, migration, and invasion of LSCC cells [35,36]. These findings highlight the potential role of XIST in HNSCC.

Enrichment analysis of the KEGG pathways and GO terms showed that the JAK-STAT signaling pathway is one of the KEGG pathways associated with downregulated DEGs. The JAK-STAT signaling pathway has roles in regulating basic cell biological behaviors such as proliferation, invasion, survival, inflammation, and immunity [37]. Abnormal JAK-STAT signaling contributes to cancer progression and metastasis [38]. HPV E6 and E7 oncoproteins are involved in JAK/STAT pathway imbalance in cervical cancer [39]. In addition, STAT3 and STAT5 proteins are key driving factors of HPV-induced malignant tumors and play important roles in the development of a series of HPV-related cancers [40]. Therefore, the JAK-STAT signaling pathway is closely associated with HPV-related HNSCC.

After protein–protein interaction analysis of DEGs, hub gene screening, and survival analysis, we found that SOCS1 expression was closely associated with the prognosis of patients with HNSCC. SOCS1 was initially thought to be a potent inhibitor of the JAK-STAT pathway, which not only has a negative role in downregulating JAK signaling, but can also modulate other signaling pathways that can stimulate STAT activation, and is involved in the progression of various cancers [41,42]. Herman et al. discovered that the SOCS1 gene was methylated and silenced in 60% of human hepatocellular carcinoma cell lines studied, indicating constitutive activation of the JAK-STAT pathway [14]. Other studies have demonstrated that elimination of SOCS1 leads to perinatal death and increases susceptibility to cancer in mice [13]. Therefore, high SOCS1 expression in HNSCC may play a significant role in inhibiting cancer development. Our survival analysis of hub genes also indicated that high SOCS1 expression was linked to a better prognosis than low expression of SOCS1, which is consistent with previous research. In the immunohistochemistry experiments, SOCS1 was more highly expressed in HPV-negative HNSCC tumor tissues compared to HPV-positive HNSCC tissues (*p* < 0.05). Other studies have also verified that SOCS1 methylation begins when HPV is positive, resulting in low SOCS1 expression in HPV-positive tumors [43]. Although high-risk HPV is an important oncogenic factor in the development of HNSCC, our clinical research affirmed that survival months increase with SOCS1 expression in same-HPV-status HNSCC, which matches the results we described above. However, patients with HPV-positive HNSCC have a higher three-year survival rate, which may be because HPV-positive HNSCC is more sensitive to radiotherapy and chemotherapy via the complete p53-mediated apoptotic response [44] and more patients are willing to receive multidisciplinary treatment [26,45] Besides, the extant literature indicated that the risk factors and demographic and tumor characteristics of HPV-positive patients differed from those of HPV-negative patients [46]. HPV-positive tumors were more likely than HPV-negative tumors to arise from the oropharynx, to be poorly differentiated, and to have basaloid features [10]. Incidentally, SOCS1 can be expressed in tumors in certain classifications, such as HPV-positive and HPV-negative in our study. These investigation may imply that HPV-positive HNSCC is a distinct clinical classification from HPV-negative HNSCC [45]. However, due to limited studies on the expression of SOCS1 in patients with HNSCC and the mechanism(s) connecting SOCS1 expression and HPV status, the significance of SOCS1 in the occurrence and development of HNSCC needs to be further studied.

The ssGSEA results showed that the level of immune cell infiltration was not significantly higher in the HPV-positive HNSCC group than in the HPV-negative HNSCC group. This may be because HPV evades the host immune response through regulating a series of molecular and cellular pathways during persistent infection, which may lead to virus-mediated immunosuppression. An immunosuppressive microenvironment is formed in the mucosal epithelium, promoting cancer progression [47]. However, there is also supporting evidence that there is immune enhancement in HPV-positive HNSCC, such as a high level of tumor immune cell infiltration and increased production of pro-inflammatory cytokines TNF-α and interferon-γ [48]. Thus, the relationship between HNSCC and HPV infection and its effect on the local immune environment and prognosis has become an important topic in oncology. Finally, targets discovered in the database using bioinformatic approaches can guide similar studies to screen potential oncogenes. This may be helpful in finding novel tumor biomarkers or therapeutic targets.

This study had some limitations. First, there was an absence of in vitro experiments to confirm upregulation and downregulation of gene expression. Second, we did not conduct any in vivo studies. These studies provide a theoretical basis for further insight into the occurrence, development, and metastatic mechanisms of HNSCC and spur some new approaches for the treatment of head and neck squamous cell carcinoma.

## 5. Conclusions

In this study, gene expression data from HPV-negative and HPV-positive HNSCCs were utilized for bioinformatics analysis, resulting in the identification of key genes (ANAPC11, BTBD6, CBLB, FBXL18, FBXO44, ITCH, KLHL3, SOCS1, JLHL11, and TRIM50) and critical pathways (JAK-STAT and phospholipase D signaling pathways). The high expression of SOCS1 in HPV-negative HNSCC is a potential biomarker that may be helpful in the prevention, diagnosis, and treatment of HPV-related HNSCC. However, the results of this study need to be validated through molecular biology studies and clinical trials.

## Figures and Tables

**Table 1:** Ten hub genes based on the DMNC algorithm using the CytoHubba plug-in.

Symbol	Official Full Name	*p* Value	log FC	Type
EREG	Epiregulin	0.656079718	0.371934432	up
PLCG1	Phospholipase C Gamma 1	0.073832163	0.198323663	up
ERBB4	Erb-B2 Receptor Tyrosine Kinase 4	0.029904345	−0.902305788	down
HBEGF	Heparin Binding EGF Like Growth Factor	0.215752146	−0.390014359	down
ZFP42	ZFP42 Zinc Finger Protein	0.727807289	0.217809524	up
CBX6	Chromobox 6	0.003652311	−0.840880952	down
NFKBIA	NFKB Inhibitor Alpha	0.453595663	0.143335238	up
SOCS1	Suppressor Of Cytokine Signaling 1	0.007229292	0.675522051	up
ATP2B2	ATPase Plasma Membrane Ca^2+^ Transporting 2	0.049796706	−1.009179634	down
CEND1	Cell Cycle Exit And Neuronal Differentiation 1	0.035879997	−0.613561026	down

## Data Availability

The datasets used and/or analyzed during the current study are available from the corresponding author on reasonable request.

## Supplementary Materials

The following supporting information can be downloaded at: https://www.mdpi.com/article/10.3390/cimb45070353/s1.
