# Supplementary figures and images for: SOCS1 as a Biomarker Candidate for HPV Infection and Prognosis of Head and Neck Squamous Cell Carcinomas
Source: Curr Issues Mol Biol. 2023 Jun 30;45(7):5598–612. doi: 10.3390/cimb45070353 (PMC10378037; doi:10.3390/cimb45070353)

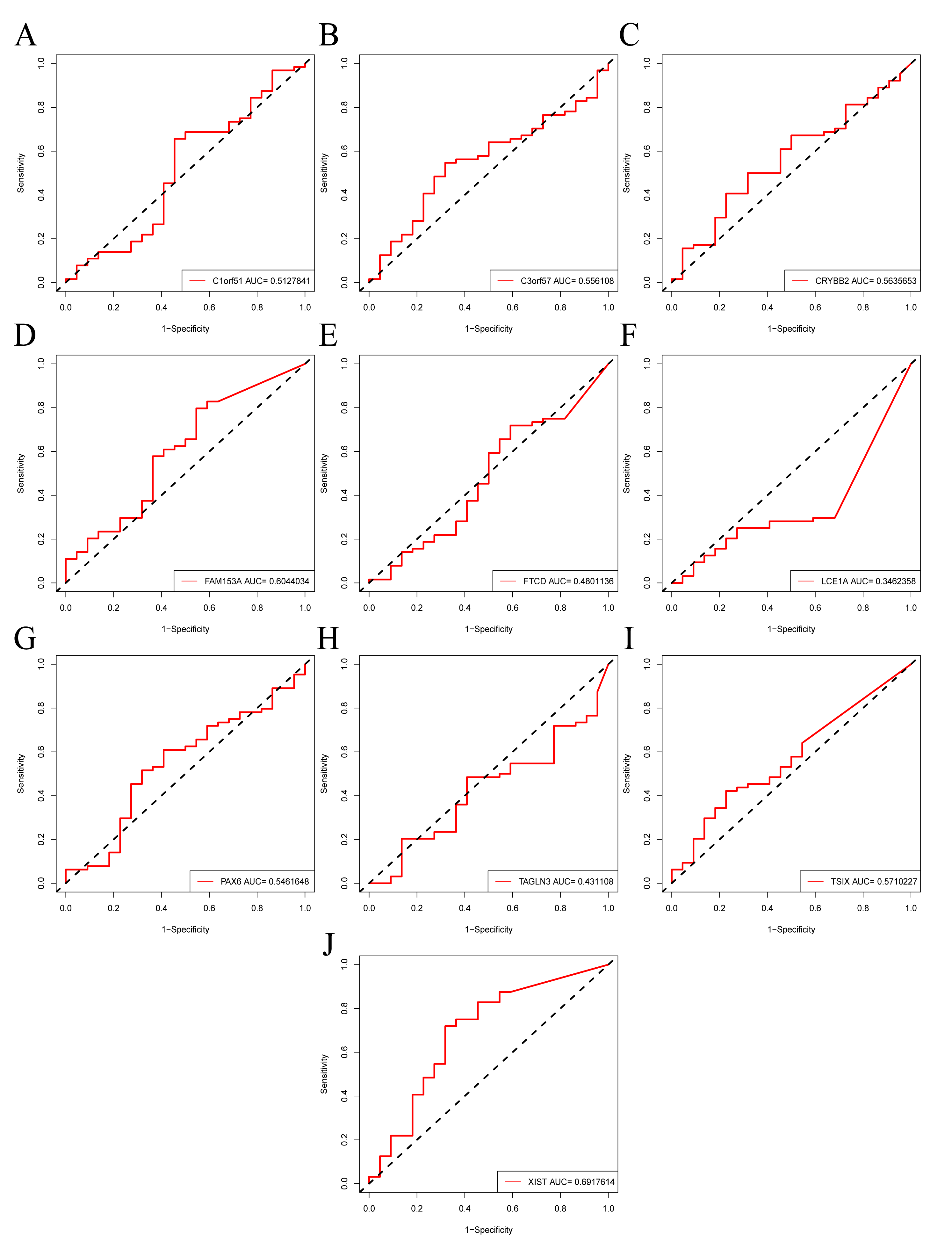

Supplement: Supplementary file 1 [file cimb-45-00353-s001.zip › Figure S1.tif]

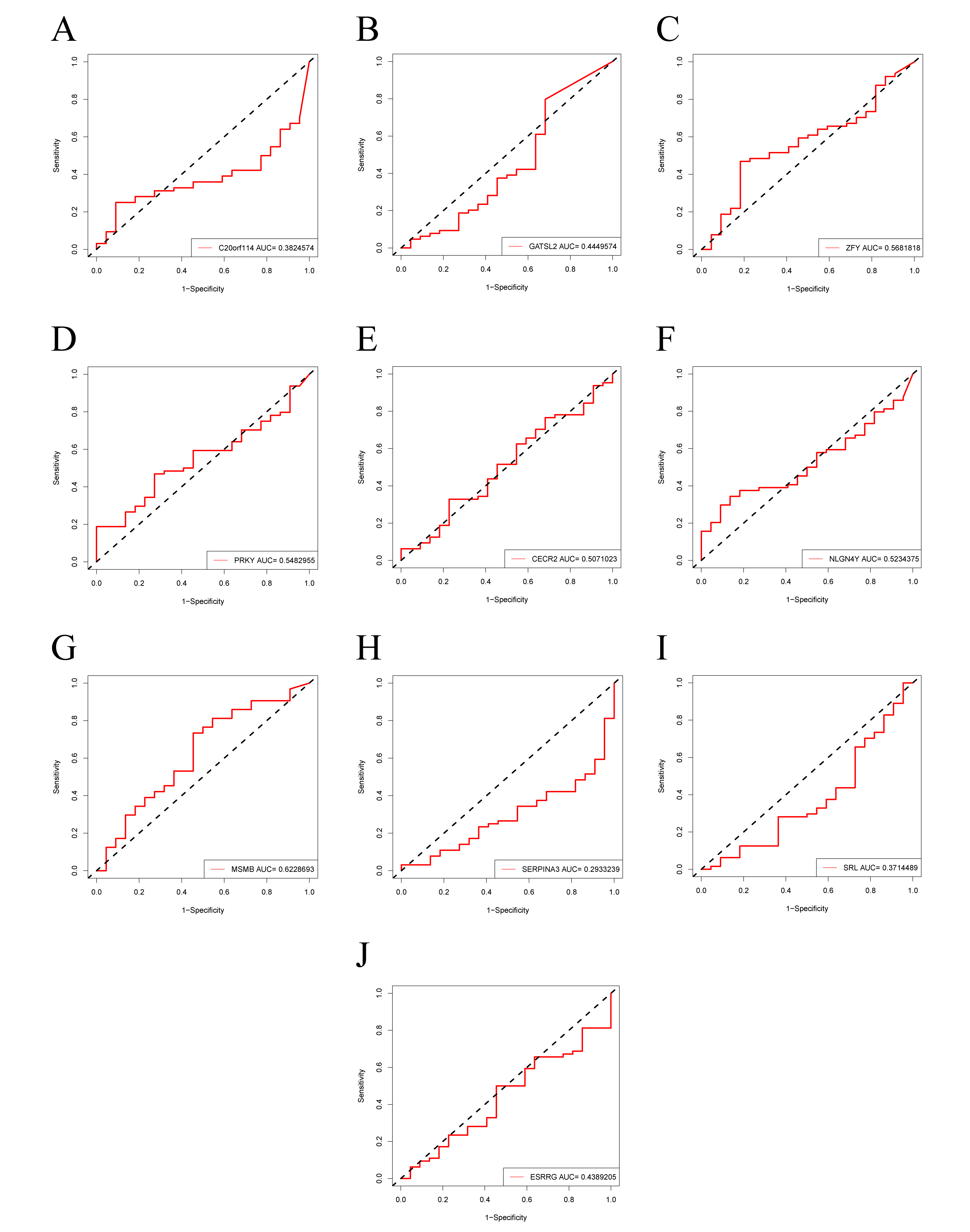

Supplement: Supplementary file 1 [file cimb-45-00353-s001.zip › Figure S2.tif]

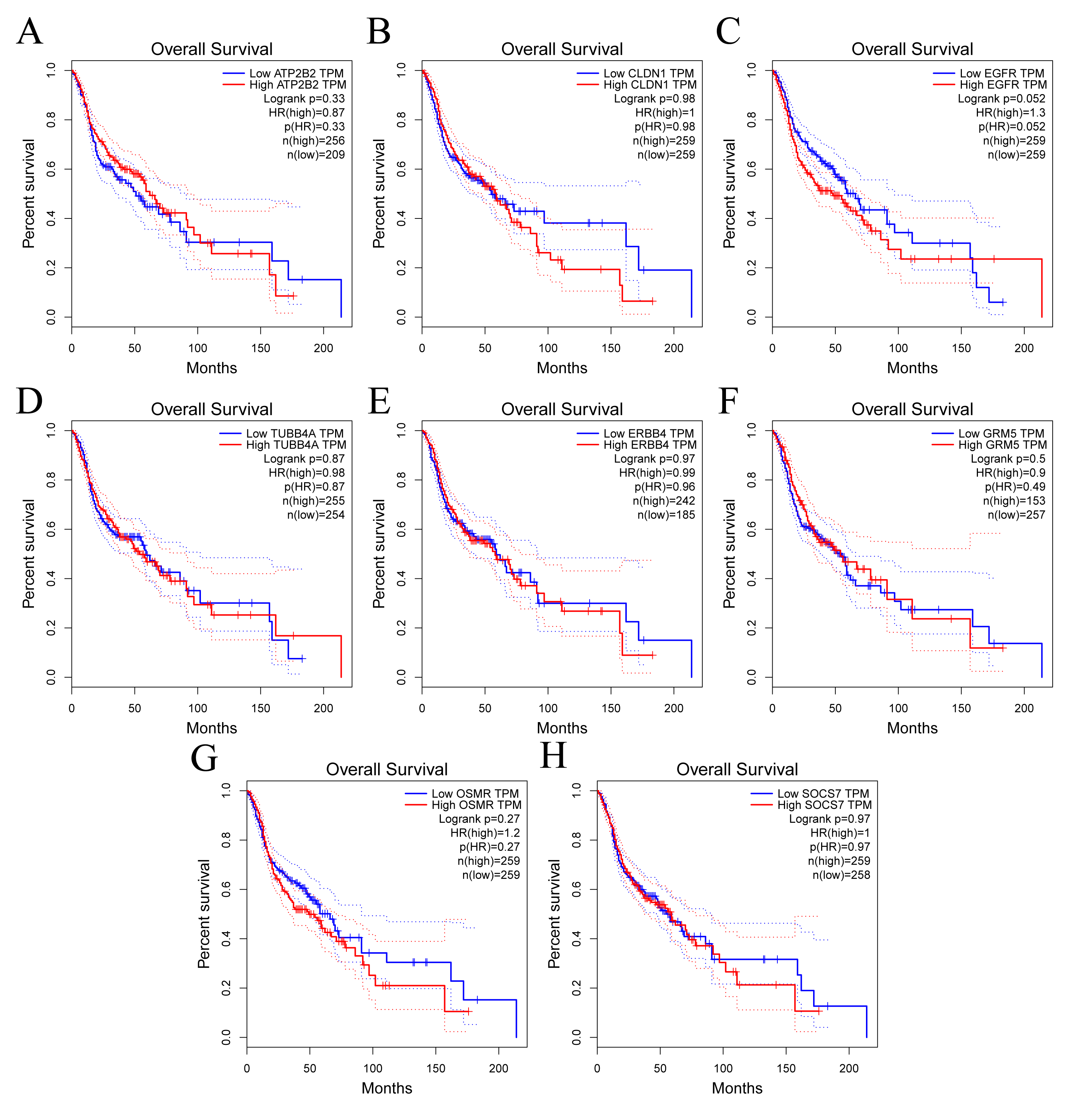

Supplement: Supplementary file 1 [file cimb-45-00353-s001.zip › Figure S3.tif]

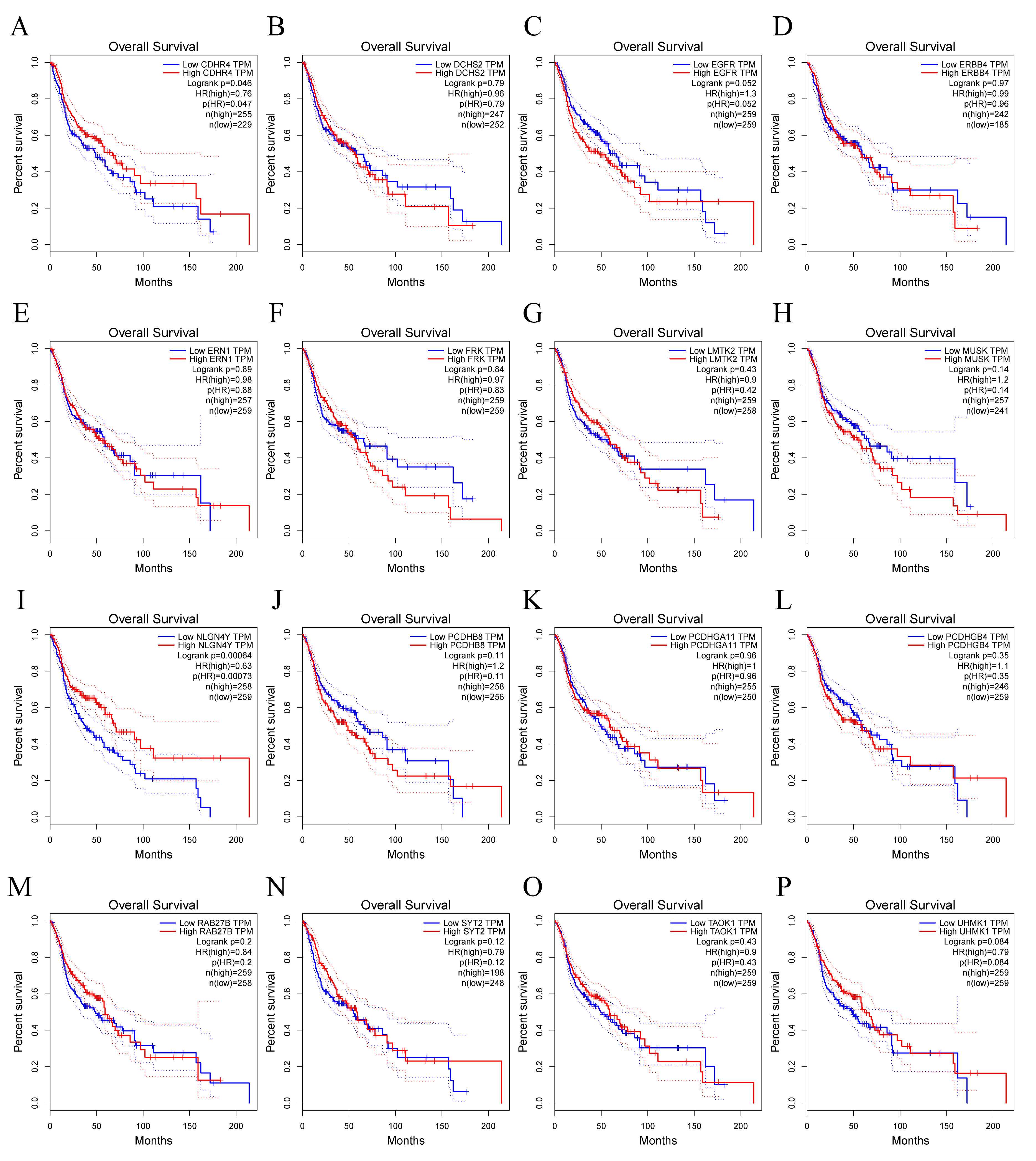

Supplement: Supplementary file 1 [file cimb-45-00353-s001.zip › Figure S4.tif]
